# Feasibility of supervised telehealth exercise for patients with advanced melanoma receiving checkpoint inhibitor therapy

**DOI:** 10.1002/cam4.6091

**Published:** 2023-05-15

**Authors:** Brendan J. Crosby, Robert U. Newton, Daniel A. Galvão, Dennis R. Taaffe, Pedro Lopez, Tarek M. Meniawy, Muhammad A. Khattak, Wei‐Sen Lam, Elin S. Gray, Favil Singh

**Affiliations:** ^1^ Exercise Medicine Research Institute Edith Cowan University Joondalup Western Australia Australia; ^2^ School of Medical and Health Sciences Edith Cowan University Joondalup Western Australia Australia; ^3^ School of Human Movement and Nutrition Sciences University of Queensland Saint Lucia Queensland Australia; ^4^ Pleural Medicine Unit Institute for Respiratory Health Perth Western Australia Australia; ^5^ Department of Medical Oncology Sir Charles Gairdner Hospital Nedlands Western Australia Australia; ^6^ Centre for Precision Health Edith Cowan University Joondalup Western Australia Australia; ^7^ Department of Medical Oncology Fiona Stanley Hospital Murdoch Western Australia Australia; ^8^ School of Medicine University of Western Australia Crawley Western Australia Australia

**Keywords:** exercise, health‐related outcomes, immunotherapy, melanoma, quality of life, telehealth

## Abstract

**Purpose:**

To determine the feasibility, safety and preliminary efficacy of a telehealth supervised exercise programme in patients with advanced melanoma receiving checkpoint inhibitor therapy.

**Methods:**

A 8‐week non‐randomised feasibility pilot trial utilising a telehealth delivered multimodal exercise programme undertaken thrice weekly with assessments at baseline and post‐intervention. The study was considered feasible if there were no severe or life‐threatening adverse events as a result of exercise, and three or more of the following criteria were met: the recruitment rate was >50%, completion rate was >80%, median programme attendance was >75%, median exercise compliance >75%, and average tolerance was >70%. Preliminary efficacy was assessed for objective measures of physical function (2‐min step test, repeated chair stand test, 30‐s push‐up test, and a modified static balance test) and quality of life (QoL), fatigue and other patient‐reported outcomes were assessed using the European Organisation for Research and Treatment of Cancer Quality of Life Questionnaire Core 30.

**Results:**

Eleven patients (32–80 years) were included in the study (6 female, 5 male). The recruitment rate was 48%, completion rate 91%, programme attendance 88%, median exercise compliance 82.1% and 84.9% for resistance and aerobic exercise, respectively, and tolerance 88%, with no severe or life‐threatening adverse events as a result of exercise. In terms of preliminary efficacy, physical function significantly improved while QoL was maintained following the intervention.

**Conclusion:**

An 8‐week telehealth exercise intervention is feasible and safe for patients with advanced melanoma and appears to improve physical function while preserving QoL during checkpoint inhibitor therapy.

## 1 INTRODUCTION

The advent and widespread adoption of systemic therapies such as immunotherapy and molecular targeted therapy have substantially improved survival rates for patients with advanced melanoma. The current 5‐year survival rate of patients with advanced melanoma receiving therapies such as programmed cell death protein 1 (PD‐1)/cytotoxic T‐lymphocyte‐associated protein 4 (CTLA‐4) inhibitors and v‐Raf murine sarcoma viral oncogene homologue B (BRAF)/mitogen‐activated protein kinase kinase (MEK) inhibitors ranges from ~34% to 52%.^1^ This is a significant improvement compared to a decade ago when the 5‐year survival rate was <10% for patients with advanced melanoma.^2^ However, grade 3 (severe) and grade 4 (life‐threatening) adverse events are common in this population during systemic treatment, especially with combination immunotherapy.^1^ Among the range of adverse events, fatigue, weakness, pneumonitis and a decreased cardiac ejection fraction have been often reported,^3^ substantially affecting quality of life (QoL) and wellbeing of patients/survivors.

Exercise has been considered an important non‐pharmacological therapy for patients during and/or following cancer treatment. Current guidelines recommend using exercise and physical activity interventions for a range of cancers including breast, prostate, colorectal, lung, haematological and head and neck cancers.^4^, ^5^, ^6^, ^7^ When utilised as adjuvant therapy within these cancer populations, exercise can alleviate a range of treatment‐related side effects such as sarcopenia, lymphoedema, metabolic syndrome, myalgia and arthralgias, in addition to improvements in physical, functional and psychological distress outcomes.^4^, ^5^, ^6^, ^7^ However, for patients with melanoma, there is a paucity of evidence as to the feasibility and benefits of exercise.^8^ As observed in a recent systematic review from our team,^8^ most studies in patients with melanoma focused on cross‐sectional/retrospective data and only a few investigated feasibility when exercise is undertaken during treatment.^8^ This indicates that the positive effects of exercise seen in other cancers are yet to be demonstrated in patients with melanoma. Concurrently, as melanoma is currently one of the few cancers for which immunotherapy is commonly prescribed, a novel opportunity exists to test exercise medicine in patients receiving this effective treatment.

The global emergence of coronavirus disease (COVID‐19) has significantly impacted traditional exercise settings and service delivery. Social distancing measures, quarantine and self‐isolation rules had been put in place to protect the population, particularly those with chronic diseases. Patients with cancer,^9^ especially those of increasing age and comorbidities,^10^ are at an increased risk of mortality if infected with COVID‐19. Accordingly, the utilisation of telehealth, that is, the use of telecommunication techniques for the delivery of health services and transmission of health information over a distance,^11^ has been suggested as an alternative method of exercise delivery for patients with cancer.^12^, ^13^ Previous studies examining telehealth exercise programmes in patients with different types of cancer (primarily breast and prostate cancer) report symptom relief and no adverse events.^14^, ^15^ Therefore, this study aimed to determine the feasibility, safety and explore the preliminary efficacy of a telehealth supervised exercise programme in patients with melanoma receiving checkpoint inhibitor therapy. We hypothesised that an exercise intervention during treatment for melanoma is feasible and does not adversely affect patient‐reported or physiological outcomes.

## 2 METHODS

### 2.1 Study design

We undertook an 8‐week non‐randomised feasibility pilot trial with assessments at baseline and post‐intervention. Intervention sessions included resistance, aerobic and balance exercises undertaken three times per week resulting in a total of 24 exercise sessions. The research project was approved by the Edith Cowan University Human Research Ethics Committee (2019‐00795‐CROSBY) and the Sir Charles Gairdner and Osborne Park Health Care Group Human Research Ethics Committee (RGS0000004232).

### 2.2 Participant recruitment

Melanoma patients receiving checkpoint inhibitor therapy were identified by attending oncology services at Sir Charles Gairdner Hospital, Fiona Stanley Hospital, and Hollywood Private Hospital, who provided them with a recruitment flyer containing information about the study. Flyers were also distributed through online melanoma support groups and at community events. Participants were screened to ensure they met the inclusion criteria: 18 years or older, diagnosed with stage III‐IV melanoma and receiving or about to receive checkpoint inhibitor therapy. Participants were excluded if they: (i) had an acute illness or musculoskeletal, cardiovascular or neurological disorder that could inhibit exercise participation; (ii) had an uncontrolled medical condition (other than metastatic cancer); and (iii) presented with a cardiovascular or pulmonary contraindication to exercise listed in the American College of Sports Medicine (ACSM) guidelines.^16^ Once deemed eligible, each participant was provided with an information letter with detailed experimental procedures, study details, associated risks and benefits. All participants provided written informed consent and obtained medical clearance from their general practitioner.

### 2.3 Exercise training programme

Exercise sessions were supervised virtually by an accredited exercise physiologist (AEP) via the online video conferencing platform Zoom (Zoom Inc). Sessions were conducted by a single exercise physiologist with up to three participants. Participants were given the option of morning or afternoon sessions at set times and could move/reschedule sessions within the same week (sessions that could not be rescheduled were recorded as missed sessions). Participants baseline exercise capability/fitness was determined based on their physical assessment scores at baseline and the AEP's clinical judgement. A Gymstick (Gymstick, Gymstick International Oy) and intervention materials (exercise information booklets and logs) were provided at no cost to participants. The exercise programme (≤60 min duration) included resistance, aerobic and balance exercises with a 5‐min warm‐up and cool‐down each session. Balance activities (i.e. side touching, heel‐toe walking and single‐leg balance), light aerobic activity and stretches were included in the warm‐up and cool‐down.

The resistance exercise component (~30 min) included a combination of exercises using bodyweight and elastic resistance (Gymstick). Based on the participants baseline exercise capability/fitness, they utilised either a black elastic band (1–20 kg) or grey elastic band (1–25 kg) on the Gymstick. Resistance exercise comprised training the major upper and lower body muscle groups with participants instructed to perform 2–3 sets of 8–12 repetitions for each exercise. A variety of exercises were utilised over the 8‐week period, for example, chest press, bent‐over row, shoulder press, biceps curl, squats, lunges and calf raise (Table S1). Six exercises were alternated weekly (two upper‐body, two lower‐body, one accessory exercise and one abdominal exercise), with autoregulation modelled on the Exercise and Sports Science Australia (ESSA) position statement for cancer and exercise to allow patients with advanced melanoma to self‐determine their intensity, frequency or duration of exercise collaboratively with the AEP.^4^ Exercises were paired where appropriate, for example, upper‐body followed immediately by lower‐body, with a 1‐min rest between sets. To ensure the progressive nature of the training programme, participants were encouraged to work at an intensity where the final repetitions of each set were noticeably difficult/fatiguing. If the participant could perform more repetitions than what was pre‐determined for that session, the intensity was increased by utilising an exercise progression or altering the resistance of the Gymstick (at the AEP's discretion) for the subsequent set or training session.

The aerobic exercise component (~20 min) initially included 5 sets of 1‐min intervals of moderate‐to‐high intensity on the spot/stationary exercises such as marching, boxing and jogging on the spot (Table S2) and progressed over the 8 weeks to 10 sets of 1‐min intervals. Participants were given 30 s of rest between intervals. Target intensity for the session was set between 12 and 15 on the 6–20 point rating of perceived exertion scale (RPE).^17^ Resistance and aerobic exercise progressions are shown in Table S3.

### 2.4 Study outcomes

Study outcomes were feasibility (i.e. recruitment and completion rates, programme attendance, exercise compliance and tolerance) and participant safety (i.e. adverse events). The intervention was considered feasible if there were no severe or life‐threatening adverse events as a result of exercise,^18^, ^19^ and three or more of the following criteria were met: the recruitment rate was >50%,^18^ completion rate was >80%,^19^ median programme attendance was >75%,^18^, ^19^ median exercise compliance (resistance and aerobic) was >75%,^20^, ^21^ and average tolerance was >70%.

### 2.5 Primary endpoints

Recruitment and completion rates were determined by the ratio of enrolled and referred patients, and the ratio of patients who completed and were enrolled in the exercise programme, respectively. Programme attendance was based on the number of exercise sessions attended out of the 24 scheduled sessions. Attendance outcomes were defined as *missed session* (i.e. missing single or two consecutive sessions), *interruption* (i.e. missing three or more consecutive sessions) and *permanent discontinuation* (i.e. loss to follow‐up).^20^

Exercise compliance was assessed for resistance and aerobic exercise separately. Resistance exercise compliance is defined as the ratio of the total volume of resistance exercise (i.e. product of sessions, number of exercises, sets and repetitions) completed to that prescribed.^20^ Dose modification was defined as any session requiring resistance exercise *dose reduction* or *escalation* (i.e. decrease or increase in number of exercises, sets, load and/or reps). For aerobic exercise, the overall volume was calculated as the total number of intervals completed, while aerobic exercise compliance was determined as the ratio of the total volume of aerobic exercise completed to that prescribed.^21^ Participants tolerance to the intervention was determined by comparing the achieved session RPE to target session RPE.

Participant safety was based upon the number of severe (hospitalisation) or life‐threatening adverse events attributable to the exercise intervention. Adverse events (including treatment‐related side effects, exercise‐related exacerbations and additional medication prescription) were recorded in a journal/log that participants kept and updated when necessary.

### 2.6 Secondary endpoints

Secondary endpoints included preliminary efficacy outcomes assessed at baseline and after the 8‐week intervention. All physical assessments and exercise delivery were conducted via a telehealth video conference by an AEP, while questionnaires (paper‐based) were completed by participants at home on the day of the assessment.^22^ Cardiovascular capacity was determined using the 2‐min step test (TMST).^23^ The standard error of measurement for the TMST is 2.7 steps.^24^ Functional performance was assessed by the repeated chair stand test.^25^ The reported coefficient of variation for the repeated chair stand is 5.6%.^26^ Upper body strength/endurance was measured using the 30‐s push‐up test.^27^ Balance was evaluated using a modified ESSA static balance test for older adults,^25^ whereby patients attempted to maintain balance for 30 s during semi‐tandem, tandem, single leg and single‐leg stance with eyes closed.

Participants' self‐reported balance confidence was determined using the Activities‐Specific Balance Confidence (ABC) Scale Questionnaire.^28^ The European Organisation for Research and Treatment of Cancer Quality of Life Questionnaire Core 30 (EORTC QLQ‐C30) was used to assess cancer‐specific QoL, functional and symptom outcomes.^29^ The Brief Fatigue Inventory (BFI) was used to assess fatigue levels before each intervention session. The mean of the nine BFI items was used to determine participants' global fatigue scores categorised as mild (1–3), moderate (4–6) or severe (7–10).^30^

### 2.7 Other measures

Demographics and clinical information were collected via an online questionnaire. Self‐reported height and body weight (at baseline and post‐intervention) were used to determine participants' body mass index (BMI, expressed as kg/m^2^). Physical activity was assessed using the International Physical Activity Questionnaire–Short Form (IPAQ‐SF).^31^ To gauge general physical activity behaviour, intervention session activity was excluded from the total. Participants were considered physically active if they met the ACSM recommendations (i.e. completing ≥150 min of moderate or 75 min of strenuous physical activity or a combination per week).^32^

All patient‐reported outcome measures were scored according to their corresponding scoring manual/recommendations, ABC Scale,^28^ EORTC QLQ‐C30,^33^ BFI^30^ and IPAQ‐SF.^34^ Higher scores on the ABC Scale, EORTC QLQ‐C30 (global health status/QoL, and functional subscales), and IPAQ‐SF (physical activity subscale) indicate improvement in participant‐reported outcomes, whereas lower scores on the EORTC QLQ‐C30 (symptom subscales), BFI, and IPAQ‐SF (sedentary behaviour subscale) indicate better results.

### 2.8 Sample size calculation and statistical analysis

We aimed to recruit 22 diagnosed stage III/IV patients with melanoma who were receiving checkpoint inhibitor therapy as this would have enabled us to detect a statistically significant change in aerobic capacity using a two‐tailed, paired *t*‐test with an effect size of 0.60, α‐level of 0.05 and statistical power (1 − β) of 0.80. Study design changes due to Covid‐19 resulted in a shortened recruitment window and, as such, only 11 participants were recruited. This number of participants gives us the ability to detect an effect size of 1.0 in a number of our secondary outcome measures such as aerobic capacity.

Statistical analyses were performed using IBM SPSS Statistics software (IBM SPSS Statistics, version 27, IBM Corp). Assumptions of normality and homogeneity of variance of residuals were tested using Shapiro–Wilk and Levene test, respectively. Participant retention rate, programme attendance, exercise compliance and tolerance were described using descriptive statistics. Normally distributed data are presented as mean ± SD and/or 95% confidence intervals (95% CI), while data not normally distributed are presented as median and interquartile range (IQR). To calculate the difference between baseline and post‐assessment values, a paired *t*‐test or Wilcoxon signed‐rank test was undertaken, as appropriate, for continuous data. A *p*‐value <0.05 was considered statistically significant.

## 3 RESULTS

### 3.1 Recruitment and completion rates

**FIGURE 1:**
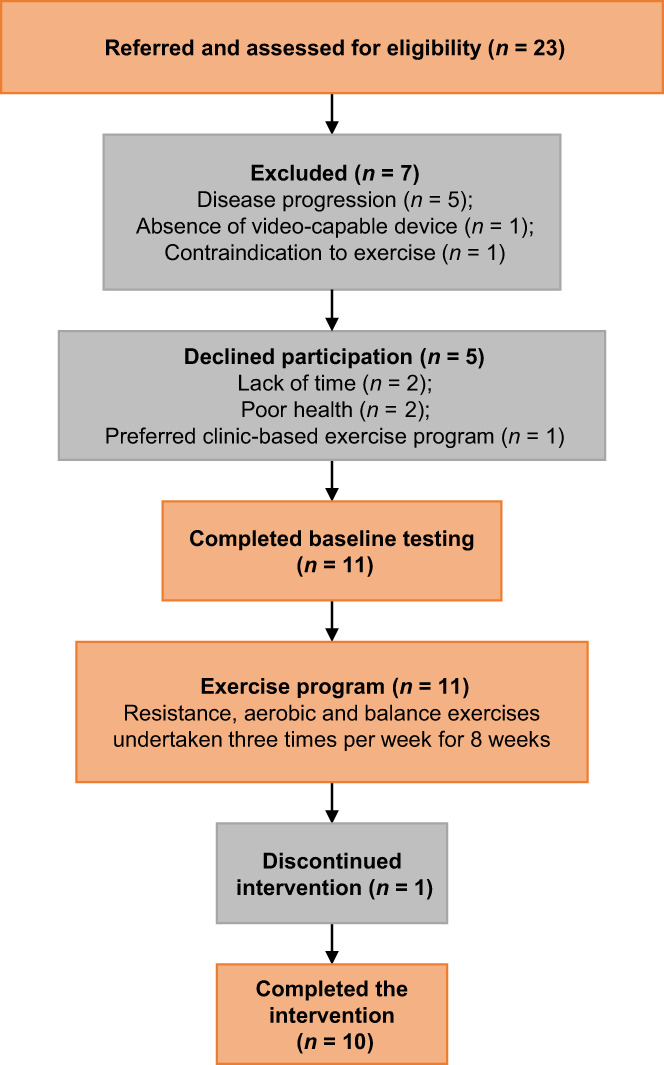
Flow chart of participant recruitment through the study.

Twenty‐three patients diagnosed with melanoma were assessed for eligibility between May 2021 and January 2022. The study recruitment rate was 48% as 11 participants consented and were recruited into the exercise programme. All 11 participants completed baseline testing. The recruitment process and loss to follow‐up are detailed in Figure 1. Seven patients (31%) were excluded from the study due to having progression of disease (*n* = 5), absence of video‐capable device (*n* = 1) and contraindication to exercise (*n* = 1). Five eligible candidates declined participation citing lack of time, poor health or preferred a clinic‐based exercise programme. Ten participants (91%) completed the 8‐week intervention, and one participant withdrew from the trial due to a severe checkpoint inhibitor treatment‐related adverse event (transient ischaemic attack).

### 3.2 Participant characteristics

Baseline characteristics are presented in Table 1. Six participants were female (54.5%), while the average age was 61.6 ± 13.6 years (range: 32–80 years). Three participants (27.3%) were physically active at baseline (i.e. ≥150 min per week) with most overweight or obese (63.8%). The majority of participants were diagnosed with stage IV melanoma (90.9%), with a median time since diagnosis of 11.0 months (IQR: 7.0–60.0 months). The most common previous treatment was surgery (81.8%). Nine participants were receiving monotherapy (PD‐1 or CTLA‐4) while two received combination inhibitors (PD‐1 and CTLA‐4). The median time since the beginning of checkpoint inhibitor treatment was 7.0 months (IQR: 0.0–9.0 months).

**TABLE 1:** Baseline demographics and clinical characteristics.

Characteristics	Participants (*n* = 11)
Demographic	
Age, mean ± SD, years	61.6 ± 13.6
Female, *n* (%)	6 (54.5%)
Married, *n* (%)	7 (63.6%)
Tertiary education, *n* (%)	2 (18.2%)
Current employed, *n* (%)	4 (36.4%)
Current smoker, *n* (%)	0 (0%)
IPAQ MET‐min/week, median (IQR)^a^	516 (363–1292)
Met PA Guidelines, *n* (%)	3 (27.3%)
Clinical	
Body weight, mean ± SD, kg	84.0 ± 18.9
BMI, mean ± SD, kg.m^−2^	28.4 ± 7.6
BMI categories, *n* (%)	
Normal weight (BMI < 25 kg.m^−2^)	4 (36.4%)
Overweight (BMI ≥ 25– < 30 kg.m^−2^)	5 (45.6%)
Obese (BMI ≥ 30 kg.m^−2^)	2 (18.2%)
Time since diagnosis, median (IQR), months	11 (7–60)
Time since treatment began, median (IQR), months	7 (0–9)
Cancer stage, *n* (%)	
Stage III	1 (9.1%)
Stage IV	10 (90.9%)
Metastasis sites, *n* (%)	
Lung	6 (54.5%)
Liver	3 (27.3%)
Lymph nodes	2 (18.2%)
Brain	2 (18.2%)
Gastric lymphoma	1 (9.1%)
Pancreas	1 (9.1%)
Spine	1 (9.1%)
Number of medications, median (IQR)	1.0 (0.0–3.0)
Comorbidities, *n* (%)	
Hypertension	3 (27.3%)
Hypercholesterolemia	4 (36.4%)
Diabetes	2 (18.2%)
Depression	1 (9.1%)
Previous treatment, *n* (%)	
Surgery	9 (81.8%)
Radiation therapy	3 (27.3%)
Chemotherapy	1 (9.1%)
Immunotherapy	4 (36.4%)
BRAF/MEK inhibitor therapy	1 (9.1%)
Checkpoint inhibitor type, *n* (%)	
PD‐1	7 (63.6%)
CTLA‐4	2 (18.2%)
PD‐1/CTLA‐4 combination	2 (18.2%)

Abbreviations: BMI, body mass index; BRAF/MEK, v‐Raf murine sarcoma viral oncogene homologue B/mitogen‐activated protein kinase; CTLA‐4, cytotoxic T‐lymphocyte‐associated protein 4; IPAQ, International Physical Activity Questionnaire; IQR, interquartile range; MET, metabolic equivalent minutes; PA, physical activity; PD‐1, programmed cell death protein 1; SD, standard deviation.

^a^ *n* = 10.

### 3.3 Programme attendance, compliance, tolerance, participant safety

The median programme attendance was 87.5% (IQR: 75.0–91.7%). Participants completed 226 out of the 264 exercise sessions scheduled. Seven participants (63.6%) missed 26 exercise sessions due to treatment‐related symptoms (*n* = 9, such as fatigue, migraine, vertigo), being unwell (*n* = 6), hospital admission (*n* = 6), psychological distress (*n* = 2) and impromptu hospital appointments (*n* = 3). One of these seven participants (participant #11; 9.1%) permanently discontinued the programme in Week 8 due to a transient ischaemic attack related to the checkpoint inhibitor treatment. Two participants (18.2%) had their exercise programme interrupted and lost six consecutive exercise sessions each due to a re‐occurrence of an axillary seroma (participant #8) and an elevated liver function test result (participant #10).

**FIGURE 2:**
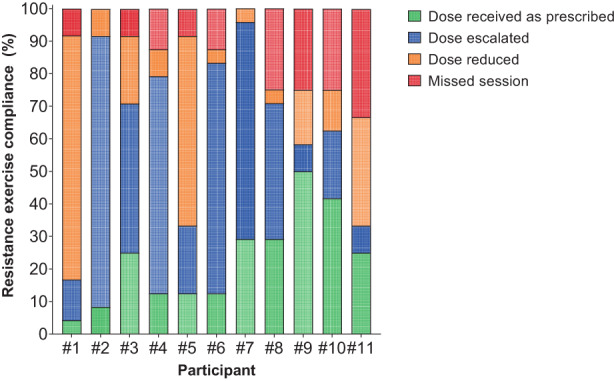
Resistance exercise compliance per participant. Data based on volume/dosage prescribed and completed.

Median exercise compliance was 82.1% (IQR: 75.3–104.3%) and 84.9% (IQR: 75.8–89.0%) for resistance and aerobic exercise, respectively. The median cumulative resistance exercise dosage completed across the intervention was 4350 repetitions (IQR: 4080–5625 repetitions). For the aerobic exercise component, a total of 1716 out of 2046 aerobic exercise intervals were completed. Exercise dose had to be modified or interrupted for 17.9% of the resistance exercise sessions. All participants had their resistance exercise programme modified at some phase of the intervention, with the exercise dose reduced or escalated in 166 out of 226 sessions completed (73.5%). Participants had a median of 3 sessions (IQR: 2–7 sessions) with the resistance exercise dose reduced (59 out of 226 sessions), while the resistance exercise dose was escalated for a median of 10 resistance exercise sessions (IQR: 4–16 sessions; 107 out of 226 sessions) (Figure 2).

**FIGURE 3:**
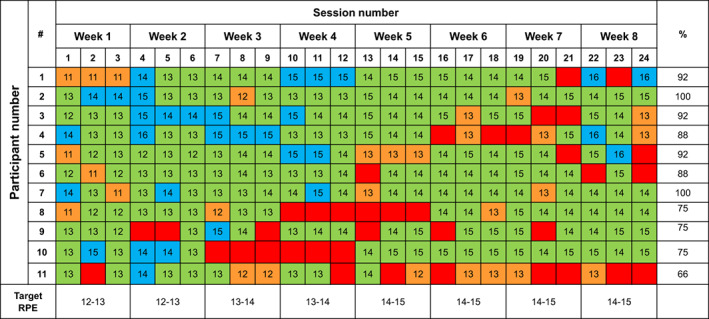
Intervention attendance and session intensity (RPE) per participant. Green squares represent that patient completed the exercise session at the prescribed rating of perceived effort; blue squares represent that patient completed the exercise sessions above the prescribed rating of perceived effort; orange squares represent that patient completed the exercise sessions below the prescribed rating of perceived effort; red squares represent missed or interrupted sessions.

With regard to exercise tolerance, 198 out of the 226 exercise sessions completed (87.6%) were performed at the prescribed (167 exercise sessions) or exceeded the target RPE (31 exercise sessions), while 28 exercise sessions were completed at a lower RPE than that prescribed (Figure 3).

**FIGURE 4:**
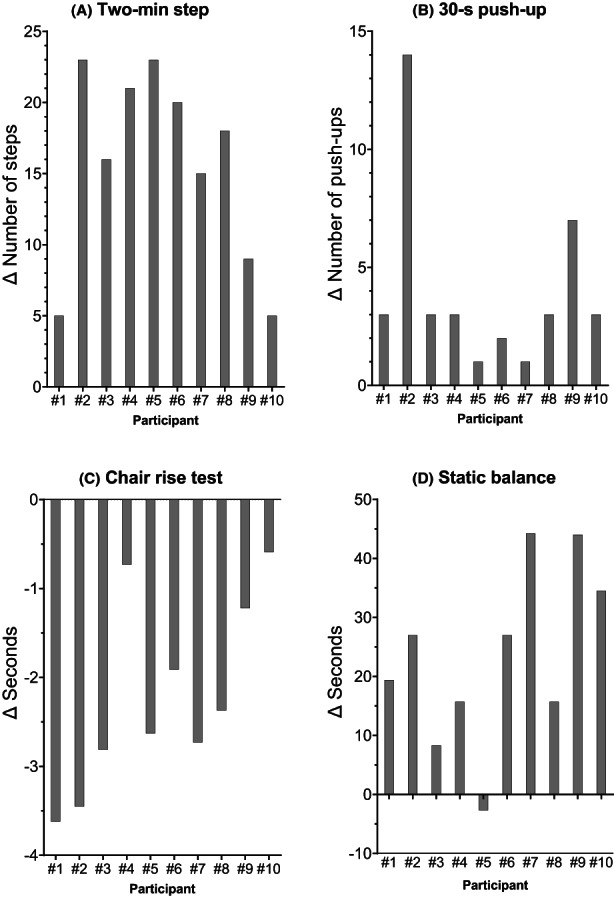
Waterfall plots of individual participants showing change in (A) 2‐min step, (B) 30‐s push‐up, (C) chair rise test, and (D) static balance over an 8‐week telehealth exercise programme. Individual patient numbers are identified in association with the bars.

No severe or life‐threatening adverse events were attributed to the exercise intervention. Adverse events are presented in Table 2. The most common checkpoint inhibitor treatment‐related side effects/adverse events were fatigue (20.0%) and diarrhoea (15.0%). Two participants (one completed the intervention, and one withdrew from the intervention) experienced a transient ischaemic attack. There was one minor exercise‐related exacerbation during the intervention sessions, which was the reopening of a surgical site (calf) 1 week after removal of suspected melanoma. In addition, one participant (participant #10 in Figures 2, 3, 4) discontinued combination PD‐1/CTLA‐4 inhibitor treatment due to an elevated liver function test result after 2 weeks into the exercise intervention. The treating oncologist suggested a 2‐week hiatus before recommencing the exercise intervention. Steroids were prescribed to manage the elevated liver enzymes and checkpoint inhibitor treatment was not recommenced before the completion of the intervention.

**TABLE 2:** Frequency of participant‐reported adverse events/side effects.

Adverse event	*n* (%)
Alopecia	1 (2.5%)
Diarrhoea	6 (15.0%)
Elevated liver enzymes	2 (5.0%)
Fatigue (moderate/severe)	8 (20.0%)
Gastrointestinal pain	1 (2.5%)
Grand mal seizure	1 (2.5%)
Hepatitis	1 (2.5%)
Hypothyroidism	1 (2.5%)
Migraine	1 (2.5%)
Nausea	2 (5.0%)
Night sweats	1 (2.5%)
Peripheral neuropathy	1 (2.5%)
Psoriasis	1 (2.5%)
Psychological distress	3 (7.5%)
Rash	4 (10.0%)
Seroma	1 (2.5%)
Surgical wound reopening^a^	1 (2.5%)
Tachycardia	1 (2.5%)
Transient ischaemic attack	2 (5.0%)
Vertigo	1 (2.5%)

^a^ A direct result of the exercise intervention.

### 3.4 Exploratory endpoints

We observed statistically significant improvements in multiple exploratory endpoints: cardiovascular capacity, upper body strength/endurance, functional performance and static balance. An increase of 15.5 steps (17.6%) was observed in the 2‐min step test (cardiovascular capacity), 4.0 repetitions (39.6%) during the 30‐s push‐up (upper body strength/endurance), 7.6 s (3.8%) in the static balance test (static balance), and a reduction of 2.9 s (23.2%) was observed in the chair rise test (functional performance) (Table 3). Individual changes in these outcomes are presented in Figure 4. There were no significant changes in global health status or any of the functional or symptom subscales following the intervention (Table 4). In addition, we did not observe improvements in perceived balance or change in body mass. The median fatigue level before each exercise intervention session was 1.9 pts (IQR: 0.9–3.8 pts). Following the intervention, a significant median increase in physical activity levels from 516 to 1374 metabolic equivalent min (MET.min) per week was observed.

**TABLE 3:** Physiological outcomes and change over 8 weeks.

Variables	Baseline	Post‐intervention	Mean difference		
	Mean ± SD	Mean ± SD	Mean	95% CI	*p*
Two‐minute step, steps	88.3 ± 27.9	103.8 ± 29.5	15.5	10.5–20.5	<0.001
30‐s push‐up, reps	10.1 ± 4.8	14.1 ± 3.6	4.0	1.2–6.8	0.010
Chair rise test, sec	12.5 ± 3.3	9.6 ± 2.8	−2.9	−4.7 to −1.1	0.006
Static balance^a^, sec	190.2 (129.3–201.3)	197.8 (160.6–219.2)	–	–	0.007

Abbreviations: 95% CI, 95% confidence interval; SD, standard deviation.

^a^ Median (interquartile range).

**TABLE 4:** Patient‐reported outcomes and change over 8 weeks.

Variables	Baseline	Post‐intervention	
	Median (IQR)	Median (IQR)	*p*
EORTC QLQ‐C30			
Global health status	66.7 (47.9–83.3)	75.0 (58.3–83.3)	0.888
Physical functioning	93.3 (65.0–100.0)	96.7 (70.0–100.0)	0.315
Role functioning	75.0 (29.2–100.0)	91.7 (62.5–100.0)	0.306
Emotional functioning	75.0 (58.3–91.7)	83.3 (58.3–93.8)	1.000
Cognitive functioning	83.3 (62.5–100.0)	91.7 (62.5–100.0)	0.279
Social functioning	66.7 (29.2–100.0)	83.3 (16.7–100.0)	0.496
Fatigue	44.4 (22.2–66.7)	33.3 (19.4–47.2)	0.440
Nausea/vomiting	0.0 (0.0–16.7)	0.0 (0.0–16.67)	0.705
Pain	25.0 (12.5–25.0)	16.7 (0.0–37.5)	0.786
Dyspnoea	0.0 (0.0–33.3)	16.7 (0.0–41.7)	0.084
Insomnia	33.3 (25.0–66.7)	33.3 (0.0–66.7)	1.000
Appetite loss	0.0 (0.0–33.3)	0.0 (0.0–8.33)	0.157
Constipation	33.3 (0.0–33.3)	0.0 (0.0–33.3)	0.046
Diarrhoea	0.0 (0.0–33.3)	0.0 (0.0–8.3)	0.705
Financial difficulties	0.0 (0.0–100.0)	33.3 (0.0–100.0)	0.157
ABC Scale, pts	140.5 (115.0–150.0)	147.0 (122.3–149.0)	0.192
IPAQ‐SF			
Physical activity levels^a^	516 (363–1292)	1374 (435–1763)	0.047
Sedentary behaviour	3225 (2363–4500)	2550 (1650–3450)	0.107

Abbreviations: ABC Scale, Activities‐Specific Balance Confidence Scale; EORTC QLQ‐C30, The European Organisation for Research and Treatment of Cancer Quality of Life Questionnaire Core 30; IPAQ‐SF, International Physical Activity Questionnaire–Short Form; IQR, interquartile range.

^a^ MET‐min/week.

## 4 DISCUSSION

The present study examined the feasibility, safety and preliminary efficacy of an 8‐week telehealth supervised exercise programme in patients with melanoma receiving checkpoint inhibitor therapy. The telehealth exercise intervention was feasible, tolerable and safe for patients with melanoma receiving checkpoint inhibitors. In addition, physical function measures were significantly improved while QoL was preserved despite patients undergoing checkpoint inhibitor therapy, well‐known to cause reductions in QoL.

This is the first study to report on supervised telehealth exercise in advanced melanoma patients or those undertaking checkpoint inhibitor therapy. Except for the recruitment rate which was just below the target of 50%, all the feasibility outcomes indicate that telehealth exercise is a feasible and safe intervention for this patient group. The telehealth exercise intervention exceeded the pre‐determined criteria for study outcomes (>3) with a completion rate of 91%, programme attendance of 88%, median exercise compliance of 82.1% (resistance exercise) and 84.9% (aerobic exercise), and tolerance at 88%, without causing any severe or life‐threatening adverse events. While two participants self‐reported experiencing a transient ischaemic attack, neither occurred during exercise and neither participant's GP attributed the event to the intervention.

It has been consistently reported that patients with advanced or metastatic cancer are under‐represented in survivorship research and services, given the complexity of their supportive care needs.^35^ In this small cohort study, we found that a short‐term telehealth exercise programme may result in meaningful physical function and balance improvements while preserving QoL in advanced melanoma patients. Our results, if confirmed by further research, could provide clinicians with information on additional tools to support these patients achieve better physical and health‐related outcomes during checkpoint inhibitor therapy. This could potentially result in a better prognosis by more effectively managing cardiovascular, metabolic and musculoskeletal health.^36^, ^37^

Moreover, delivering exercise through telehealth is also a novel aspect of the study. A number of health‐related services have had to be changed during the COVID‐19 pandemic,^38^ including access to supervised exercise. Most cancer patients have limited access to health services due to distance, transport, inconvenience, and financial capacity, resulting in an unacceptable disparity and suboptimal QoL for those patients who cannot access the best practice in melanoma care. Although our results need to be confirmed with a larger randomised controlled trial, the telehealth supervised exercise programme implemented in the present study is an important strategy to remove the disadvantage of patients unable to access clinic‐based facilities due to financial or geographic constraints.^38^ Additionally, previous telehealth exercise interventions in a variety of cancer populations have reported symptom relief without causing severe adverse events.^14^, ^39^, ^40^ Interestingly, even with an expected demanding cancer treatment regime due to a higher number of consultations, we observed that exercise intervention completion rates, programme attendance and exercise compliance were moderate to high, resulting in a relatively high tolerance of this group of patients. This may be partially explained by the supervised telehealth component of the intervention, enabling participants to complete sessions in their chosen location without the need to travel or attend a physical venue. Therefore, our data support future studies examining how telehealth exercise can improve treatment‐related outcomes in patients with advanced melanoma and, ultimately, be part of future exercise recommendations for cancer patients.

Our findings on preliminary efficacy may be important given the association between cardiorespiratory fitness and muscle strength with independent living and survival in older patients and those living with cancer.^41^, ^42^, ^43^, ^44^ Additionally, increased aerobic capacity, lower body muscle power, upper body strength and balance are associated with decreased fall risk among older adults.^45^, ^46^ These observed gains may represent the translation of exercise effects to potential health benefits in this group of patients, however, we must consider the small sample size. Also, our finding that body weight was maintained during the intervention is important as reductions in body weight during checkpoint inhibitor therapy are related to poorer survival.^47^ Exercise programmes involving resistance training (i.e. anabolic exercise) might prove effective in this context, through maintenance of body weight while increasing lean body mass (i.e. muscle mass).^48^, ^49^, ^50^ Therefore, using resistance‐based exercise programmes through telehealth may be a potential strategy to reduce the risk of sarcopenia and cachexia^51^, ^52^ for advanced melanoma patients.

Since advanced melanoma patients receiving checkpoint inhibitor therapy can have lower QoL than matched controls,^53^ managing patient‐reported symptoms and outcomes is an important aspect of holistic health care. Among the potential mediators, intense treatment^54^ and an advanced tumour^55^ are associated with reductions in QoL. Exercise has been shown to significantly improve QoL when treating various cancer types.^56^ Our exploratory analysis on QoL aligns with that of the Lacey et al.^57^ study, which reported patients with melanoma receiving checkpoint inhibitor therapy preserved QoL over an 8‐week supportive care intervention that included exercise, dietary advice, psycho‐oncology services and complementary therapies. Similar to the study of Lacey et al.,^57^ the small sample size of the present study may limit the ability to detect meaningful change in this outcome. Nevertheless, maintaining QoL would still benefit advanced cancer patients. Interestingly, it has been suggested that advanced cancer patients may prioritise QoL over the length of life when receiving cancer treatment, potentially even refusing treatment to maintain QoL.^58^ Maintaining QoL with exercise is feasible and a low‐cost intervention, which may improve treatment adoption and reduce treatment modification/cessation.

The strength of the present study is that it comprised a structured multimodal design (i.e. aerobic, resistance and balance activities) for patients with advanced melanoma receiving checkpoint inhibitor therapy. In addition, the utilisation of telehealth to provide supervised exercise is particularly relevant for individuals with advanced cancer (potentially immuno‐compromised), enabling access to exercise services for patients in metropolitan, rural and remote settings. The information provided regarding exercise metrics such as attendance, exercise compliance and tolerance enables the replication of this study on a larger scale. However, some limitations are worthy of comment. Changes to hospital recruitment procedures (following COVID‐19) and disease progression among many patients with advanced melanoma made recruitment difficult; consequently, our sample size was small, and a control group was not utilised. Moreover, due to the COVID‐19 pandemic and patients advanced melanoma status, assessments were undertaken via telehealth. As a result, the learning effect within the physiological testing outcomes could not be measured without a non‐exercise control group, potentially contributing to the changes in physical function observed. Given the number of comparisons/analyses undertaken, we cannot discount that at least one of the significant findings may be due to chance. Future studies should utilise a randomised controlled design to further investigate the effects on QoL and other patient‐reported symptoms during a longer exercise intervention. Additionally, we recommend that future studies examine the effect of exercise on muscle mass/body composition during checkpoint inhibitor treatment using techniques such as dual X‐ray absorptiometry. Finally, monitoring treatment compliance/tolerance during an exercise intervention will provide important clinical insights.

In conclusion, an 8‐week online telehealth exercise intervention is feasible and well tolerated by patients with melanoma receiving checkpoint inhibitor therapy with no major adverse events. Further, the intervention appeared to improve physical function in this group of patients while preserving QoL. These are important findings to inform the design of future randomised trials, which should include larger patient numbers, a usual care control group, a longer exercise intervention, and objective measures of body composition.

## AUTHOR CONTRIBUTIONS

**Brendan J Crosby:** Conceptualization (lead); data curation (lead); formal analysis (lead); investigation (lead); methodology (lead); project administration (lead); software (lead); validation (lead); writing – original draft (lead); writing – review and editing (lead). **Robert U. Newton:** Conceptualization (supporting); investigation (supporting); methodology (supporting); supervision (equal); validation (supporting); visualization (supporting); writing – review and editing (supporting). **Daniel A Galvao:** Conceptualization (supporting); investigation (supporting); methodology (supporting); supervision (equal); validation (supporting); visualization (supporting); writing – review and editing (supporting). **Dennis R Taaffe:** Conceptualization (supporting); formal analysis (supporting); investigation (supporting); methodology (supporting); supervision (equal); validation (supporting); visualization (supporting); writing – review and editing (supporting). **Pedro Lopez:** Conceptualization (supporting); data curation (supporting); formal analysis (lead); investigation (supporting); methodology (supporting); software (supporting); visualization (supporting); writing – review and editing (supporting). **Tarek Meniawy:** Conceptualization (supporting); methodology (supporting); supervision (supporting); validation (supporting); visualization (supporting); writing – review and editing (supporting). **Muhammad A Khattak:** Conceptualization (supporting); methodology (supporting); supervision (supporting); validation (supporting); visualization (supporting); writing – review and editing (supporting). **Wei‐Sen Lam:** Conceptualization (supporting); methodology (supporting); supervision (supporting); validation (supporting); visualization (supporting); writing – review and editing (supporting). **Elin S. Gray:** Conceptualization (supporting); investigation (supporting); methodology (supporting); supervision (equal); validation (supporting); visualization (supporting); writing – review and editing (supporting). **Favil Singh:** Conceptualization (supporting); data curation (supporting); formal analysis (supporting); investigation (supporting); methodology (supporting); project administration (supporting); supervision (lead); validation (supporting); writing – review and editing (supporting).

## CONFLICT OF INTEREST STATEMENT

No financial support was received to conduct the present study, or for the preparation or publication of this manuscript. Sponsors were not involved in the study design, analysis or interpretation of data, manuscript writing and decision to submit the manuscript for publication.

## ETHICS STATEMENT

The research project was approved by the Edith Cowan University Human Research Ethics Committee (2019‐00795‐CROSBY) and the Sir Charles Gairdner and Osborne Park Health Care Group Human Research Ethics Committee (RGS0000004232).

## Supporting information


Table S1.
Table S2.
Table S3.

## Data Availability

The data that support the findings of this study are available from the corresponding author upon reasonable request.
