# Supplementary material for: Feasibility of supervised telehealth exercise for patients with advanced melanoma receiving checkpoint inhibitor therapy
Source: Cancer Med. 2023 May 15;12(13):14694–706. doi: 10.1002/cam4.6091 (PMC10358269; doi:10.1002/cam4.6091)
Supplement: Supplementary file 1 — Table S1. Table S2. Table S3. [file CAM4-12-14694-s001.docx]

**Table S1.** Resistance exercise intervention and planned exercise regressions and progressions.

| **Standard Resistance Exercise** | **Resistance Exercise Regression** | **Resistance Exercise Progression** |
| --- | --- | --- |
| Squats | ¼ Squats | Back Squats (Gymstick) |
| Push ups | On Wall/Chair/Knees | Feet Elevated |
| Calf-Raise | - | Single Leg/Gymstick on Shoulders |
| Upright Row | Seated High Pull | - |
| Triceps Extension | - | - |
| Supine Lying Leg lowers | Supine Lying Leg Slides | - |
| Chest Press | Seated Chest Press (Gymstick Behind Chair) | - |
| Lunges | ¼ Lunges (Chair for Support) | Gymstick on Shoulders |
| Bent-Over Row | Seated Row | - |
| Biceps Curl | Seated Biceps Curl | - |
| Plank | Plank Against Chair/Wall | - |
| Chest Fly | Seated Chest Fly (Gymstick Behind Chair) | - |
| Seated Leg Extension | - | - |
| Rear Deltoid (Wide Pull) | - | - |
| Shoulder Press | Seated Shoulder Press (Seated on Gymstick) | - |
| Wall Sit | ¼ Wall Sit | - |
| Standing Gymstick Core Rotations | Seated Gymstick Core Rotations | Russian Twist (Seated Trunk Rotation) |
| Kneeling Rear Leg Extension (Kick) | Standing Rear Leg Extension (kick) | - |

**Table S2.** Aerobic exercise intervention with planned exercise regressions and progressions.

| **Standard Aerobic Exercise** | **Aerobic Exercise Regression** | **Aerobic Exercise Progression** |
| --- | --- | --- |
| Marching | Seated Marching | Marching with opposite arm raise (climbing ladder) |
| Standing Boxing | Seated Boxing | Shadow Boxing |
| Side Steps with Arm Lifts (Star Steps) | Seated Star Jumps | Star Jumps |
| Kettlebell Swing (no weights) | ¼ Squat Chair Touch and Reach High | Full Squat Ground Touch and Reach High |
| Air Squats | Sit and Stand from Chair | Jumping Squats |
| Basic Burpee (without Push ups) | Knee/Wall Push ups | Burpees |
| Side Steps with Butt Kicks | Side Steps | Skaters |
| Butt Kicks | Stepping on the Spot | Running on Spot |
| Marching | Seated Marching | High Knees |
| Standing Elbow to Knee | Seated Elbow to Knee | Air Ladder Climb |

**Table S3.** Resistance and aerobic exercise planned progression throughout the study period.

|  | **Weeks 1-2** | **Weeks 3-4** | **Weeks 5-6** | **Weeks 7-8** |
| --- | --- | --- | --- | --- |
| **RT (Sets/Reps)** | 2 sets of 10 reps | 2 sets of 8 reps  to  3 sets of 12 reps | 3 sets of 10 reps  to  3 sets of 12 reps | 3 sets of 8 reps  to  3 sets of 10 reps |
| **AE Intervals** | 5-6 sets of 1 min | 7-8 sets of 1 min | 8-9 sets of 1 min | 9-10 sets of 1 min |
| **Target Session RPE** | 12 - 13 | 13 - 14 | 14 - 15 | 14 - 15 |

Abbreviations: RT, resistance exercise; AE, aerobic exercise; RPE, rating of perceived exertion.
